# Improving the Ability to Write Persuasive Texts in a Boy with Autism Spectrum Disorder: Outcomes of an Intervention

**DOI:** 10.3390/brainsci10050264

**Published:** 2020-04-30

**Authors:** Sergio Melogno, Maria Antonietta Pinto, Andrea Ruzza, Teresa Gloria Scalisi

**Affiliations:** 1 Department of Psychology of Development and Socialization Processes, “Sapienza” University of Rome, 00185 Rome, Italy; mariantonietta.pinto@uniroma1.it (M.A.P.); ruzza.andrea@gmail.com (A.R.); gloria.scalisi@uniroma1.it (T.G.S.); 2 Faculty of Psychology, “Niccolò Cusano” University of Rome, 00166 Rome, Italy

**Keywords:** persuasive text writing, perspective-taking, autism spectrum disorder, adolescence, intervention

## Abstract

In this paper, we describe an intervention implemented to assist a 13.2-year-old boy with Autism Spectrum Disorder, G, without intellectual disability, aimed at improving his ability to compose persuasive texts. There was an initial assessment (baseline), an intermediate assessment after two weeks, a six-session intervention phase, and a post-intervention assessment. Our intervention applied two procedures. The first aimed at enhancing general composition abilities in terms of picking (P) ideas, organizing (O) notes, and writing (W) them down (POW), while the second specified the steps to write a persuasive text addressing a possible reader: a topic sentence (T), reasons (R), an explanation (E) for the reasons and the end of the sentence (E) (TREE). These procedures were termed POW + TREE. To analyze G’s texts, three types of measures were used by two raters at baseline, intermediate and post-test time: (a) the presence of the TREE components; (b) the quality of the reasons and explanations for the reasons; (c) the number of mental state terms. All these measures showed relevant quantitative improvements, as well as qualitative changes. In addition, when G’s performance at the end of the intervention was compared to that of typically developing controls, no statistical difference appeared. The results are discussed in light of the potentialities offered by the type of intervention described here.

## 1. Introduction

Autism Spectrum Disorder (ASD, henceforth) is an umbrella expression that designates a set of heterogeneous early onset neurodevelopmental conditions. In general terms, these conditions are characterized by well-known patterns of specific behaviors during social interaction and communication, and unusually restricted and repetitive activities and interests [1]. Prognostic studies suggest better outcomes in individuals with ASD who possess a higher intellectual level, relatively fluent language at the beginning of primary school, and reduced difficulties in social abilities. Actually, follow-up studies show a plurality of developmental trajectories in children with ASD (for a review, see Lai, Lombardo and Baron-Cohen [2]).

In the learning area, this clinical population, even without intellectual disability, generally shows heterogeneous profiles. For instance, in a study conducted on 100 adolescents (mean age: 15.6) by Jones and colleagues [3], in terms of reading and mathematics competence, the authors found “peaks and dips” in the profiles of the participants. In every participant, there was at least one ability that was markedly over or under the expected level. In a similar vein, Randi, Newman and Grigorenko [4], who reviewed studies on the profiles of readers with ASD, found that these profiles were extremely variable.

In the area of writing, children with ASD shown similar heterogeneous skills (see Zajic and Wilson [5]). Some children with ASD show well-developed writing skills and may even become skillful writers [6], while others manifest difficulties that place them below their typically developing peers’ levels [5,7]. In a recent meta-analysis, Finnegan and Accardo [7] identified six critical components in the writing abilities of individuals with ASD compared with their typically developing peers, namely handwriting length, legibility, size, speed, spelling and structure, while no difference appeared in sentence construction. The factors that might account for differences in these critical components are still under study. Nevertheless, according to Accardo and colleagues, and Zajic and Wilson [5,8], it is highly plausible that differences in the texts produced by individuals with ASD are related to Theory of Mind [2], executive function, fine motor skills and/or speech and language skills. Another source of variability could be associated to the type of texts these individuals are faced with. In particular, persuasive text seems to be one of the most difficult [9] due to the following reasons. 

As the goal of a persuasive text is to persuade a reader about the value of some arguments, overcoming all possible counter-arguments, the writer’s concern is to argue his/her opinions on a given topic, provide reasons to support these opinions, and defend them. To sum up: composing a persuasive text requires the writer to adopt the interlocutor’s point of view and revert it by using even stronger arguments. To this end, the interlocutor’s arguments must be taken into account but also overridden by further, undisputable arguments. All these operations make the writing of a persuasive text a particularly sophisticated communicative task. 

In particular, a persuasive text requires competence in the very object of debate, turn-taking ability as a component of Theory of Mind, an ability to weigh the various facets of the issue at hand, an ability to construct an appropriate synthesis of both the arguments and the counter-arguments, which, in turn, requires integrative processing skills [2]. In addition, there are also linguistic requirements such as appropriate vocabulary, particularly concerning mental states (epistemic and emotional–volitional words and expressions), inter- and intra-propositional cohesion, and knowledge of typical rhetorical devices in writing. For instance, a persuasive text must contain connectives such as “that is” (to be precise), “indeed” (to present evidence), “therefore” (to draw conclusions). Lastly, the sequence of statements is guided essentially by logical, rather than temporal criteria, which entails the use of other types of connectives (“in addition”, “as a consequence”, “in summary”, “overall”, “in conclusion”) [9,10,11]. 

Brown, Johnson, Smith and Oram Cardy’s [11] study, based on two groups of adolescents, one of 25 students (mean age: 12), with ASD but without linguistic impairment, and another group of 22 typically developing students (mean age: 13), apparently supports the above hypothesis. The participants had to read a series of directions aimed at writing persuasive texts on a screen. The main differences between the two groups concerned all production measures (examples the number of words), lexical and syntactic complexity, quality of the arguments, but not cohesion measures and writing conventionalities. The authors interpreted the lower quality of the texts produced by the participants with ASD in light of Flower’s concept [12] of “writer-based text”, as opposed to “reader-based text”. The former was thus termed because it does not take into account the reader’s perspective and is characterized by two main features: insufficient integration between components into a higher-order framework, which results in lists of details instead of a general concept, and insufficient clarity, due to over-vague and ambiguous referencing.

In more recent years [8,9,13], there has been a growing interest in the multiple procedures, often used in combination, which adults can apply to support the writing process in individuals with ASD. Concerning persuasive text, certain types of interventions have specifically aimed at inducing a shift from a writer-based perspective to a reader-based perspective, as indicated by Brown and colleagues’ study [11]. These authors suggested: (a) the use graphic organizers as tools to support the planning phase (pre-writing activities); (b) to teach how to graduate from factual details to higher-order concepts; (c) to teach participants how to weigh the strength of each individual argument as a basis for organizing the whole argument; (d) to provide participants with visual supports to recall the various steps of the writing process; and (e) to encourage students to ask for feedback from readers [11]. All these suggestions are also mentioned in the research synthesis by Accardo and colleagues [8]. Asaro-Saddler and Saddler [14], and Asaro-Saddler and Bak [15], investigated the possibility of enhancing this type of writing using the Self-Regulated Strategy Development (SRSD) program, which was originally developed by Graham and Harris [16,17]. This program aims at teaching planning, stimulating a flexible use of strategies, and promoting both a positive attitude towards writing and a positive self-image as a writer. This study implemented two lessons and mnemonics that were also implemented in Asaro-Saddler and Bak [15]. The first aimed at enhancing general composition abilities in terms of picking (P) ideas, organizing (O) notes, and writing (W) them down (POW), while the second specified the steps to write a persuasive text addressing a possible reader: a topic sentence (T), reasons (R), an explanation (E) for the reasons and the end of the sentence (E) (TREE). The participants were three children with ASD, between eight and nine years old. The authors compared three persuasive baseline essays with three post-intervention texts and found evident improvements, both in qualitative and quantitative aspects, which gives support to the effectiveness of the POW + TREE approach. Asaro-Saddler [18], after reviewing 11 studies investigating the specific strategy of SRSD used in the writing instruction of learners with ASD, found that these students improved their planning ability, the number of written elements, and the content of their writing when using the self-regulated strategy.

In our study, we applied a program with a boy, conventionally called “G”, with ASD and without intellectual disability, but with clear difficulties in writing persuasive texts, as attested by his teachers. In this article, we will consider G’s change over six persuasive writing tasks: two at baseline, two after two weeks, and two after the intervention. In addition, we also considered the performance of a control group of typically developing children in the last two persuasive writing tasks, written at the same point in the school year, and compared G’s performance to that of the controls. While an increase in G’s overall performance was expected as a function of the intervention, it was more difficult to foresee whether there would be differences, and in which areas these differences might be found, between G’s performance and that of the controls who had not undergone any intervention at all regarding persuasive text composition. 

## 2. Materials and Methods

To evaluate the outcomes of the intervention implemented with G, we analyzed his change in text composition, starting from an initial assessment (baseline), followed by an intermediate assessment after two weeks, itself followed by a six-session intervention, and lastly by a post-intervention assessment. We also compared the last phase of G’s production to the persuasive text composition of typically developing children (*n* = 8) enrolled in the same school grade (mean age: 13.5 at G’s post-test time). The study was approved by the Ethics Committee of the Department of Developmental and Social Psychology, “Sapienza” University of Rome. Informed consent was given freely by G’s and the controls’ parents.

### 2.1. Participants

G was a 13.2-year-old boy at the beginning of the intervention, and was enrolled in grade 8 in an Italian public school. He had been diagnosed as a child with ASD without intellectual disability. A first diagnosis, based on DSM-IV-TR [19], was then confirmed on the basis of the DSM 5 criteria [1] at the age of 11. The instruments used were the Autism Diagnostic Observation Schedule—Second Edition (ADOS—2) [20], an interview with G’s parents and teachers, focused on the social and communicative aspects of G’s behavior, and his learning profile.

In the social area, there was a clear discrepancy between G’s interaction abilities with adults or with peers, with the former being more adequate. Among the reasons that made G’s interaction with his peers problematic, we must mention his difficulty in perspective-taking, his erudite language, and over-developed moral rigidity. G also developed specific knowledge on some topics, most often felt by his interlocutors as too sophisticated and cultivated. For instance, faced with a given conflict between his friends, G responded with a political reference, mentioning the Foreign Affairs Ministry and the Internal Affairs Ministry, using the corresponding metonymical expressions (“Farnesina” (the British equivalent of “Farnesina” would be “Downing Street”) for the former, and “Viminale” for the latter). Lastly, in everyday routines, G would show some dysfunctional behavioral patterns that appeared difficult to modify.

As mentioned above, G’s intellectual level, as measured by the Intellectual Quotient of the Wechsler Intelligence Scale for Children, 4th edition (WISC-IV) [21], was average (IQ: 107) although it was not representative of his performance across each index: Verbal Comprehension Index (126); Perceptual Reasoning Index (124), Working Memory Index (85), Processing Speed Index (71). In contrast, G’s General Ability Index (128), which is based on the first two indices, can be considered as representative. It must be noted that the weighted score for Vocabulary (18), based on word definition, places G in the very above average range. His grammatical comprehension score (108), as measured by the Test for Reception of Grammar, 2nd edition (TROG 2) [22], was also average. His sentence production abilities were very good (z: 1.45), as measured by Gugliotta and colleagues’ test [23], as well as his verbal reasoning (z: 1.17), measured by the same test, which assesses the capability to identify absurd statements in sentences, understand proverbs, identify a super-ordinate category, and differences in word pairs. 

In his academic abilities, G showed some strengths and weaknesses. For instance, in a standardized Italian reading test [24], his comprehension performance was adequate both for accuracy and speed, reaching the 90th percentile. In contrast, G’s handwriting appeared slow, as measured by an Italian test [25] that evaluates writing speed, subdivided into three parts, each of which are performed in one minute: (a) writing the two graphemes “l” and “e” in italics in a continuous way; (b) writing the Italian word “uno” (Eng: “One”) as many times as possible; (c) writing as many words designating numbers as possible. The z scores were as follows: (a) within norm; (b) z: −1.43; (c) z: −2.09. Despite the above praxic difficulties in writing, G’s teachers reported that the child was perfectly able to compose descriptive and narrative texts using the font he had better automatized, namely capital script, while he was very poor at composing persuasive texts.

The controls were recruited randomly in the same classroom as G’s in a school attended by families sharing the same sociocultural background, without learning disabilities nor any other type of developmental disorder. The whole classroom had followed a standard school curriculum, without a specific focus on argumentative text as in the program implemented with G. Based on teacher’s quantitative assessment, the controls’ performance in persuasive texts ranged from adequate to good, and for this reason we did not assess their competence in this type of text at baseline.

### 2.2. Intervention Procedures

Our intervention focused on teaching two mnemonics implemented in earlier research studies with children with ASD [15]. The first aimed at enhancing general composition abilities in terms of picking (P) ideas, organizing (O) notes, and writing (W) them down (POW), while the second specified the steps to write a persuasive text addressing a possible reader: a topic sentence (T), reasons (R), an explanation (E) for the reasons and the end of the sentence (E) (TREE). As an extension of the explanation category, which represents the core of the argumentation process, we also considered counter arguments (C.Arg), i.e., those arguments that are in favor of the reader’s perspective. We will therefore describe the activities implemented in each session not as an abstract schema, but as the actual sequence applied to G’s case, namely: modeling (1), joint writing (2), guided writing (3), autonomous writing (4). Therefore, the POW + TREE procedures were applied flexibly as a function of G’s reactions.

Each session lasted about 90 min. In the first session (modeling), the adult illustrated the aim of the session and interactively analyzed the meaning of the expression persuasive text (PT), defined as: “A PT tells the reader what the writer believes or thinks about a particular topic”.

The adult explained and modeled POW and TREE using a thinking-aloud procedure. In the planning phase (P), the adult would make the following type of suggestion: “To write my text, the problem must be very clear in my mind…I must have some idea about the topic…and therefore, I might have to search for information in books, on the Internet, or ask the others, etc…”. In the organization phase (O), the adult would say: “To convince the reader that my opinion is a valid one, I must organize my thoughts in a logical manner: first, I will state the problem at hand and my personal opinion…then, I will give my reasons and try to explain them the best I can…and, in the end, I will draft a conclusion”.

In the writing phase (W) the adult would say: “Now I am going to write the text based on the POW I wrote before”. In this process, he would call into question his linguistic choices: “Will the reader understand my idea the way I phrased it?”, and justify them: “Maybe here it’s better to write “indeed”, because I wish to prove my reasons”, etc.

**Figure 1:**
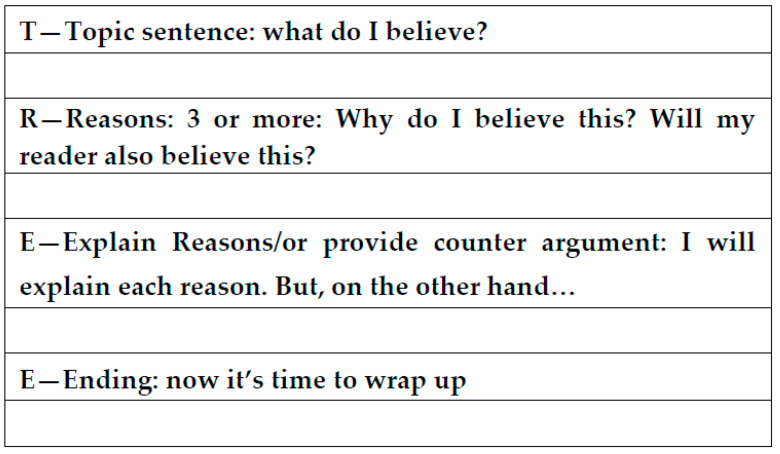
Topic sentence (T), reasons (R), explanation (E) and end of the sentence (E) (TREE) graphic organizer—Graham and Harris. Adapted from Asaro-Saddler and Bak [15] with some modifications.

In this session, the adult composed a PT on how to persuade a frequent video game user not to stick to the screen at the expense of more constructive activities, such as social exchange with friends or reading interesting things. In order to model the second TREE component (R), the adult argued that video games can cause severe addiction (R1); that not every video game is as stimulating as other games in real life (R2); that excessive video-game playing can impoverish one’s social life with peers (R3). Once the text was complete, the adult and G identified the TREE components and transferred them into the graphic organizer (Figure 1).

At the beginning of the second session (joint writing—first step), the adult showed G the two texts he had written during the baseline phase and invited him to identify the TREE components. To facilitate the task, the adult asked G to fill the slots of the graphic organizer (Figure 1) and, in relation to each component, G wrote the action that best fit the meaning of it.

In the third session (joint writing—second step), the adult chose a text from a book and analyzed the TREE components jointly with G in order to check G’s comprehension. Afterwards, the adult and G interactively wrote a further PT text. The adult supported G during the writing process, in particular when the child omitted something important, and also mitigated G’s negative thoughts, such as: “I really have no idea”, “I really don’t see how to do that”. To contrast the child’s negative feelings, the adult used self-reinforcing sentences, such as: “I remember once I could overcome more difficult obstacles than this”; “In the future, what I am doing now might prove helpful”. G had a list of these self-reinforcing sentences, named the “thought chart”, which he could consult at any time.

**Figure 2:**
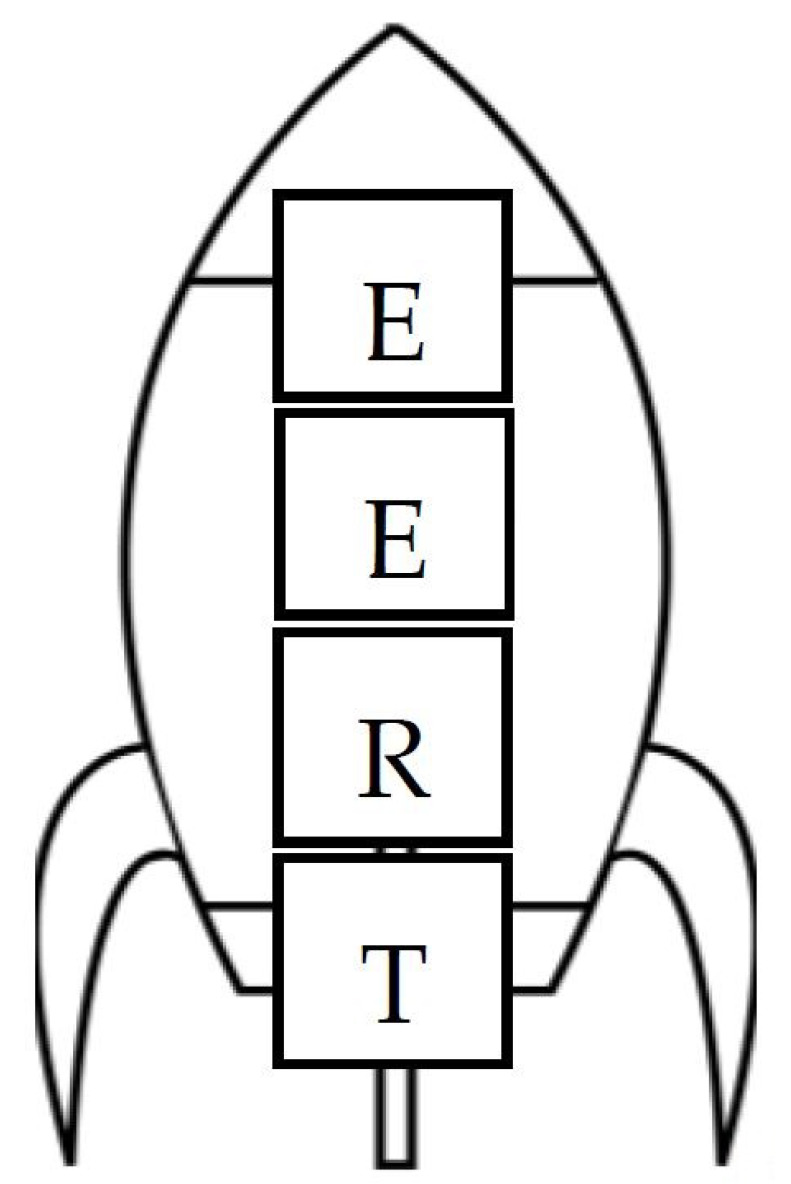
The rocket image to check the presence of the TREE components.

In the fourth session (guided writing—first step), G had to fill up his TREE graphic organizer by himself. The adult did not intervene anymore regarding the text, but stimulated G to follow the various steps of the POW procedure, and encouraged him to use the thought chart. At the end of the composition, G was invited to check the presence of all the components in his text, as represented by the image of a rocket (Figure 2). In other words, G was told that the “rocket” could not start if the components were not all present. In the fourth session, G produced two PTs.

In the fifth session (guided writing—second step), G did not need to base his writing on the TREE graphic organizer anymore, because he had already memorized it, and rehearsed the POW procedure by himself. The adult just reminded G to use the thought chart. In this fifth session, G produced two PTs.

In the sixth and last session (autonomous writing), G wrote two PTs in a totally autonomous way, sometimes rehearsing the POW procedure by himself and recalling some of the self-reinforcing sentences of the thought chart.

### 2.3. Measures

To analyze the outcomes of the intervention in terms of the level of the PTs produced (G was allowed to choose the font he had better automatized, which was capital script), a series of criteria were applied, partly inspired by Asaro-Saddler and Bak’s study [15], blending quantitative and qualitative aspects. The topics of the PTs had been chosen based on the interests that motivated G, as reported by his parents, and those which could be shared with his peers: the use of mobile phones, McDonald’s restaurants, holidays at the seaside, bad experiences with animals, reasons for not going to school during summer and the use of social networks. The following is an example of directions for composing a persuasive text: “Your friend had a bad experience: he was bitten by a dog. From that moment onwards, he did not want to have contact with any kind of animal anymore. Try to write a text to convince him to approach the animal world again”.

Two independent raters, who did not know the nature of the intervention nor the sequence of the text composition, analyzed the six texts written by G and the two texts written by the controls on the basis of three criteria: (1) the presence/absence of the TREE components (topic, reasons, explanation/counter argument, ending); (2) the qualitative level of the reasons and explanations/counter arguments; (3) the amount of mental state terms.

For the first criterion, the scores varied depending on the components. For topic and ending the scoring was dichotomous: the absence of a topic or ending was worth no points, and the presence of topic or ending was worth one point. For reasons and explanations/counter arguments, the score varied as a function of the number of reasons or explanations/counter arguments provided by the participants. For example, one reason or one explanation/counter argument was worth one point, two reasons or explanations/counter arguments were worth two points, etc.

As for the second criterion, the qualitative level of reasons and explanations/counter arguments, a four-point scale (zero to three) was applied, one for each criterion separately. For reasons, a score of zero was attributed to no reasoning or irrelevant reasoning; a score of two was attributed to ill-focused reasoning, a score of two was attributed to relevant but non exhaustive reasoning, and a score of three was attributed to exhaustive reasoning. For explanations/counter arguments, a score of zero was attributed to no explanation or irrelevant explanation/no counter argument or irrelevant counter argument; a score of one was attributed to ill-focused explanation/ill-focused counter argument; a score of two was attributed to relevant but non exhaustive explanation/relevant but non exhaustive counter argument, and a score of three was attributed to exhaustive explanation/exhaustive counter argument.

To assess the third criterion, i.e., the amount of mental state terms, the rater had to identify and count two categories of words or expressions: epistemic and emotional–volitional. The score resulted from the total number of these words or expressions in each text.

- Epistemic verbs: “I know/I don’t know; I think/I don’t think; I believe/I don’t believe”, etc.;
- Epistemic locutions: “it seems to me; to me”, etc.;
- Epistemic nouns: an idea; a thought; an opinion, etc.;
- Emotional–volitional verbs: I like; I do not like; I want, etc.;
- Emotional–volitional nouns: pleasure; disgust, etc.;
- Emotional–volitional adjectives: marvelous; horrible, etc.

## 3. Results

Table 1 reports the scores of all the measures considered in G’s PTs. We can observe that the scores related to the presence/absence of the TREE components increased from five to six from the initial to the intermediate phase and then markedly improved up to 20 in the post-test and, in particular, in the final PT, where G gave four reasons and four explanations. As for the scores assessing the qualitative aspects of reasons and explanations/counter arguments, there is an almost exponential improvement: 2–4–18 for the reason scale, and 0–5–20 for the explanations/counter arguments scale.

The scores assessing the separate amount of epistemic and emotional–volitional terms, and the total amount of mental state terms also show a very relevant improvement, where the total score increases from six, to 11, and then to 23, despite some discrepancies between epistemic and emotional–volitional terms in some areas.

To compare G’s performance in the last two PTs to the performance of the controls in the same PTs we applied Crawford and Howell’s [26] method, used to compare an individual with control samples that have modest N (e.g., <10). According to this method, the statistics of the control sample are treated as sample statistics, rather than as population parameters, and the *t*-distribution (with *n*—1 degrees of freedom) is used, rather than the standard normal distribution, to evaluate the abnormality of the individual’s scores. In this modified *t*-test procedure, the p value represents the probability of individuals in the population from which the normative sample was drawn of obtaining a score as low as that observed for the individual.

Crawford and Howell’s [26] method was applied to all the measures described in Table 2 (*t* values and two-tailed probabilities are reported in brackets). As the standard deviation of topic and ending was zero in the control group, it was impossible to perform the comparison with G’s scores. For all the other measures, no significant differences were found.

## 4. Discussion

In this article, we described an intervention implemented with a 13.2-year-old boy with ASD, G, without intellectual disability, aimed at improving his ability to compose persuasive texts, a pragmatic–linguistic ability that was clearly poor according to his teachers. This weakness was particularly striking in light of G’s erudite language and cultivated comments. His refined references to political institutions, presented as possible methods of interpreting very common social interactions, made his discourse difficult to understand, especially to his peers.

Our design included an initial assessment (baseline phase), an intermediate assessment after two weeks, a six-session intervention phase, and a post-intervention assessment. The intervention drew on Asaro-Saddler and Bak’s study [15], where Self-Regulated Strategy Development [16,17] was applied to enhance the writing of PTs. In our study, the POW + TREE intervention program was implemented in six sessions, subdivided into four phases: modeling, joint writing, guided writing and autonomous writing. To analyze the six texts considered in this study, three types of measures were used by two raters at baseline, intermediate and post-test time: (a) the presence/absence of the TREE components; (b) the quality of the reasons and explanations for these reasons and/or counter arguments; (c) the number of mental state terms.

The score assessing the presence of the TREE components increased from five to six from the baseline to the intermediate phase and then, quite remarkably, up to 20 in the post-test. A similar trend was attested in the growth of the quality of both reasons and explanations. Reasoning scores increased from two to four from the baseline to the intermediate phase and then, abruptly, to 18 at post-test, while explanations/counter argument scores increased from zero to 5 from the baseline to the intermediate phase, and then to 20 in the post-test. We believe the first increase might be attributed to the topic of PT4, centered on the use of the mobile phone, a particularly attractive one for G. In general terms, the nature of the contents most probably influenced the overall performance. However, this factor alone can hardly account for the transition from the initial texts, where the TREE structures were nearly absent, to the texts at the end of the intervention where these structures were very salient. In addition, we could observe a noticeable growth in the use of mental state terms, mainly on the epistemic side, i.e., from six words and expressions at the baseline, to 11 at the intermediate phase, to 23 in the post-test. It must be noted that, before the intervention, G’s mental state terminology was present but unevenly distributed (see PT3 vs. PT4), while, at post-test, it became both richer and better distributed (PT5 and PT6).

If we consider the balance between the structural aspects of the texts in terms of the presence of the TREE components, and the qualitative aspects represented by mental state terms, we can better understand the nature of G’s growth. In the very first text (PT1), the TREE structure is partial and mental state terms are rather poor and are only constituted by the use of epistemic words. At the other extreme, in PT6, the TREE structure is not only complete, but is also based on high-level explanations, and, concomitantly, the mental state terminology reaches its maximum, with a balance between epistemic and emotional–volitional expressions.

What deserves attention, in our view, is that these improvements in both structural and lexical aspects match a psychological shift, from a writer-based- to a reader-based perspective, following Brown and colleagues [11]. In other words, G showed good linguistic resources in his first persuasive text, but he did not use these resources to persuade a hypothetical interlocutor. At the end of the intervention, G put these resources at the service of a reasonment based on representations, his own and the others’. It is probable that the above psychological shift was provoked at a precise phase of the intervention, namely the joint writing phase. For the first time, G had to compare his point of view with that of hypothetical others, with an argumentative aim in mind and, to this end, he had to choose well-focused words. At the same time, the joint writing practice paved the way to the autonomous writing phase, where G was stimulated to produce texts on his own, and apply all the devices he had been taught. This gradual transition from joint writing to G’s autonomous production marks the transition from hetero- to self-regulation.

The following example (PT5, reactions after a dog bite), illustrates G’s ability to analyze the different facets of the same issue and ponder the validity of arguments and counter-arguments. “……Even after a dog’s bite, one can approach the animal world again (Topic). Not every animal behaves like dogs (R1). It has been a very rare accident (R2). The animal world has much to offer” (R3). Visibly, G provided a skilled argument and counter argument of the topic: (a) there are different categories of animals, which may differ in behavior; (b) a bite is not, per se, an absolute event and thus it cannot be generalized as a bad behavior; (c) animals are also capable of highly valuable behaviors. In addition, G supported his reasons with very appropriate explanations: “For instance, a tortoise or a rabbit are much less aggressive than a dog (in relation to R1). Moreover, dogs sometimes bite while playing (in relation to R2). People do love their pets because they offer them strong emotions: tenderness, love, friendship” (in relation to R3). On conceptual grounds, we must note how acute G’s counter arguments are and, linguistically, how finely he can modulate his thoughts. In addition, we must remark that these conceptual and linguistic means serve a socio-cognitive function: the hypothetical friend’s point of view is reverted into a convincing new perspective. Finally, in the ending, G recapitulated his argument in a very elegant register: “Therefore, even if you had an unpleasant accident, you should give a *second chance* (our italics) to the animal world!”. Therefore, G included the act of biting in the broader category of an “accident”, and categorized the invitation to try another approach with animals in more abstract terms, namely those of a “second chance”, which he underlines with a significant exclamation mark.

Comparing G’s performance in the post-test with his peers’ produced text showed no significant differences. Therefore, G’s best performance was close to that of his typically developing peers, at least on quantitative terms. Nevertheless, on qualitative grounds, we cannot help noticing that the controls organized both the initial presentation of the topic and the final recapitulation of the arguments in a more elaborate way. These children would characterize the very scenario of the hypothetical dialogue in detail, so as to render the whole argumentation process more plausible. In the ending, they would recapitulate more systematically the pros and cons, arguments and counter arguments with explicit reference to the “other’s point of view”. Another aspect that deserves attention is the extensive use of rhetorical devices—in particular, metaphors, idioms, proverbs and humor [27,28]—generally reported as a weakness in some individuals with ASD [27,28,29,30,31]. For example, in PT6, (topic: going to fast food restaurants), we found the following examples from different participants of the control group. “Non è tutto rose e fiori” (English: “It’s not all fun and games”) (before introducing a counter-argument). “…riempiono (i fast-food) di felicità le papille gustative” (…“they (fast-foods) fill taste buds with happiness” (this is a totally unconventional metaphorical usage in Italian)). “Affogare la fame con patatine ed hamburger” (“To drown hunger with hamburger and chips” (also a totally unconventional metaphorical usage in Italian)). Both these metaphors are totally unconventional in Italian. Lastly, we will mention the frequency of explanations based on thoughts, opinions, and mental states. Ex: “Going to fast-foods; to get free from thoughts…” “…to get relaxed and distract oneself”; “There you can exchange ideas with your friends, which will help you taking important decisions”; “…to take your mind off”.

We believe this study presents some strengths. The teaching of POW + TREE followed a rigorous methodology, which was, however, flexibly implemented according to G’s reactions, phase by phase. A short, but well-articulated, intervention let written argumentative abilities emerge in a child who was perceived by his teachers as particularly poor in this type of writing. We must also point to some methodological weaknesses and possible perspectives for future research. First of all, although we could rely on teachers’ evaluations, we made no initial assessment of the controls’ capabilities. Secondly, follow-up testing should be applied to check the solidity of the results obtained by G. Thirdly, a future step of the present study could focus on G’s capability of producing other persuasive texts in the school context in order to check the generalizability of the outcomes obtained at the end of the intervention described here. Finally, we could consider the whole range of persuasive texts produced during the same lapse of time in both an individual child treated with the same type of intervention as G, and a control group. This would allow us to better grasp, beyond overall outcomes, the different trajectories of subjects with ASD, and those of typically developing children in completing this type of task. Although a case study based on a child with the characteristics of G cannot reflect the heterogeneous world of all individuals with ASD without intellectual disability, we believe it can shed light on the potentialities offered by the type of intervention described here.

## 5. Conclusions

In our study, we highlighted that writing a persuasive text involves abilities that go far beyond an academic task because they presuppose and, at the same time, stimulate the capability to think about the other’s point of view in relation to one’s own. We believe this capability represents an important form of reciprocity that can improve the subject’s adaptive functioning.

## Figures and Tables

**Table 1:** Scores of all the measures in G’s persuasive texts (PTs) in all phases.

	Baseline		Intermediate		Post-Test	
	PT1	PT2	PT3	PT4	PT5	PT6
Topic	0	0	0	0	1	1
Reasons	2	1	2	1	3	4
Exp/C.Arg.	1	1	1	2	5	4
Ending	1	0	0	0	1	1
Total PT	3	2	3	3	10	10
Total Phase	5		6		20	
Reason 1	0	0	2	2	3	2
Reason 2	2	-	-	-	3	2
Reason 3	-	-	-	-	3	2
Reason 4	-	-	-	-	-	3
Exp/C.Arg 1	-	0	1	2	3	3
Exp/C.Arg 2	-	-	-	2	3	3
Exp/C.Arg 3	-	-	-	-	2	3
Exp/C.Arg 4	-	-	-	-	0	3
Exp/C.Arg 5	-	-	-	-	0	-
Total Reasons	2		4		18	
Total Exp/C.Arg	0		5		20	
Epist	3	0	9	1	6	9
Em–Vol	0	3	1	0	4	4
Tot Epist	3		10		15	
Tot Em–Vol	3		1		8	
Tot Ment St Terms	6		11		23	

Legend: persuasive text (PT); explanations/counter arguments (Exp/C.Arg); epistemic terms (Epist); emotional–volitional terms (Em–Vol); mental state terms (Ment St terms).

**Table 2:** Comparisons between G and control scores.

	G’s Scores (*z*-Scores)	Controls’ Mean (SD)	*t*	*p*
Reasons	7 (−1.68)	9.13 (1.27)	−1.58	0.16
Exp/C.Arg.	9 (−0.33)	9.50 (1.50)	−0.31	0.76
Total	20 (−1.07)	22.63 (2.45)	−1.01	0.34
Reasons’ levels	18 (−1.79)	25.00 (3.91)	−1.69	0.14
Exp/C.Arg levels	20 (−1.51)	27.63 (5.05)	−1.42	0.20
Epistemic terms	15 (−0.69)	18.75 (5.40)	−0.66	0.53
Em–Vol terms	8 (−1.65)	17.50 (5.74)	−1.56	0.16
Total	23 (−1.36)	36.25 (9.72)	−1.29	0.24
